# Pathogeneses of respiratory infections with virulent and attenuated vaccinia viruses

**DOI:** 10.1186/1743-422X-4-22

**Published:** 2007-02-27

**Authors:** Daisuke Hayasaka, Francis A Ennis, Masanori Terajima

**Affiliations:** 1 Center for Infectious Disease and Vaccine Research, University of Massachusetts Medical School, Worcester, MA 01655, USA

## Abstract

**Background:**

Respiratory infection with the neurovirulent vaccinia virus (VV) strain Western Reserve (WR) results in an acute infection of the lung followed by dissemination of the virus to other organs and causes lethality in mice. The mechanisms of lethality are not well-understood. In this study, we analyzed virus replication and host immune responses after intranasal infection with lethal and non-lethal doses of VV using the WR strain and the less virulent Wyeth strain.

**Results:**

The WR strain replicated more vigorously in the lung and in the brain than the Wyeth strain. There were, however, no differences between the virus titers in the brains of mice infected with the higher lethal dose and the lower non-lethal dose of WR strain, suggesting that the amount of virus replication in the brain is unlikely to be the sole determining factor of lethality. The WR strain grew better in primary mouse lung cells than the Wyeth strain. Lethal infection with WR strain was associated with a reduced number of lymphocytes and an altered phenotype of the T cells in the lung compared to non-lethal infections with the WR or Wyeth strains. Severe thymus atrophy with a reduction of CD4 and CD8 double positive T cells was also observed in the lethal infection.

**Conclusion:**

These results suggest that the lethality induced by intranasal infection with a high dose of the WR strain is caused by the higher replication of virus in lung cells and immune suppression during the early phase of the infection, resulting in uncontrolled virus replication in the lung.

## Background

Vaccinia virus (VV) is a member of the *Poxviridae*, which constitute a large family of enveloped DNA viruses and replicate entirely in the cytoplasm of the infected cells with a linear double-stranded DNA genome of 130–300 kilo base pairs [1]. Poxviruses have a broad range of eukaryotic hosts including mammals, birds, reptiles and insects [2,3] and can grow in many cell lines in vitro. Some poxviruses are causative agents of human diseases. Variola virus caused a deadly human disease smallpox until its global eradication in 1977 [1,4,5], in which VV was used as a vaccine. Other poxviruses causing human diseases are molluscum contagiosum virus and the zoonotic monkeypox virus [6,7]. Notably, variola and monkeypox viruses are transmitted to humans by respiratory route, whereas molluscum contagiosum virus is mainly transmitted through the skin. Variola and monkeypox viruses cause systemic infections with high levels of lethality, but the details of their pathogenesis are not well-understood.

Intranasal inoculation of different VV strains in mice shows different levels of virulence and only neurovirulent strains cause lethality [8]. Western Reserve (WR) strain was generated by intracerebral mouse passages, and an intranasal inoculation results in an acute infection of the lung followed by dissemination of the virus to various organs [8-11]. Intranasal infection with a low dose of WR strain induces an inflammatory infiltrate in the lung, and the virus was cleared 10 to 15 days after infection [10]; however, infection with a high dose of WR strain caused lethality, which has been used as a challenge model to study the effect of antiviral drugs, immune IgG, soluble viral proteins and other vaccine strains [9,12-20]. In one report intranasal infection with the WR strain caused pneumonia showing severe alveolar edema and acute necrotizing bronchiolitis and peribronchiolitis as well as neutrophilic infiltrates in the interstitium of the lung [21]. The mechanisms of lethality in mice infected with the lethal dose of WR strain are, however, not well-understood.

In this study, we focused on the differences in virus replication and host immune responses between lethal and non-lethal respiratory infections with VV. We used two VV strains; neurotropic virulent WR strain and the less virulent Wyeth strain. Although BALB/c mice are frequently used for intranasal challenge of vaccinia virus [10,22], we used the C57BL6/J strain of mouse in these experiments for two reasons. One is that most knockout mice lacking genes involved in immune responses have been made with C57BL6/J genetic background. The other is that we and one other group have characterized cellular immune responses, especially CD8^+ ^T cell responses, to vaccinia virus in C57BL6/J mice [23,24], when this study was planned. Infection of C57BL/6J mouse with a high dose (10^6 ^p.f.u. (plaque-forming units)) of the WR strain was lethal, whereas a high dose (10^6 ^p.f.u.) of Wyeth strain and a lower dose (10^4 ^p.f.u.) of WR strain were not lethal. The WR strain replicated and produced higher titers of virus in the lung and the brain compared to the Wyeth strain. There was, however, no difference between the virus titers in brains of mice infected with the high or low dose of WR strain. Lethal infection with WR strain resulted in fewer lymphocytes and an altered phenotype of T cells in the lung compared to non-lethal infection and uninfected controls, and induced severe thymus atrophy with a marked reduction of CD4 and CD8 double positive (DP) T cells.

## Results

### Virulence of WR and Wyeth strains by intranasal infection

**Figure 1:**
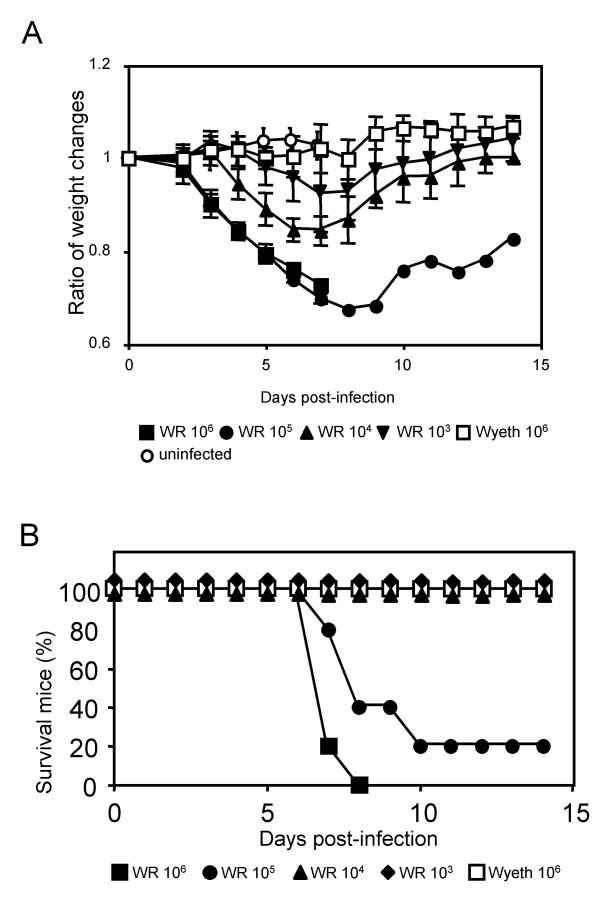
**Virulence of WR and Wyeth VV strains in C57BL6/J mice after intranasal infection**. Groups of five mice were infected with 10^6 ^(■), 10^5 ^(●), 10^4 ^(▲) and 10^3 ^(▼) p.f.u. of WR strain or 10^6 ^(□) p.f.u. of Wyeth strain, or uninfected (○). (A) Time course of the average body weight change of each group is presented. The average weight at day 0 is set as 1. The error bars indicate the standard deviations. (B) Survival curve of the infected mice in each group.

Adult female C57BL6/J mice were inoculated with various doses of the WR and Wyeth strains intranasally. Weight change and survival of infected mice were recorded daily. Inoculation with higher doses (10^6 ^p.f.u. and 10^5 ^p.f.u.) of the WR strain induced rapid and severe weight loss, which became obvious at 3 days post-infection (Fig. 1A), and most of mice died at 7–10 days post-infection (with 10^6 ^p.f.u. all mice died by 8 days post-infection) (Fig. 1B). Lower doses (10^4 ^p.f.u. and 10^3 ^p.f.u.) of the WR strain caused mild weight loss, and all mice survived. These mice recovered their weight after 6–8 days post-infection (Fig. 1A). The 50% lethal dose (LD_50_) of WR strain was calculated as 4.2 × 10^4 ^p.f.u., which is similar to the LD_50 _reported for BALB/c mouse [22]. Wyeth strain did not kill mice (Fig. 1B) or cause weight loss (Fig. 1A) even when 10^6 ^p.f.u. of the virus was inoculated.

### Virus replication in the lung and the brain

We compared virus replication in the lung and the brain. It had been reported that after intranasal inoculation, virus was recovered from various organs in mice, but higher titers of the virus were detected in the lung and the trachea [10,22]. Since WR strain is neurotropic, we also measured virus titers in the brain.

**Figure 2:**
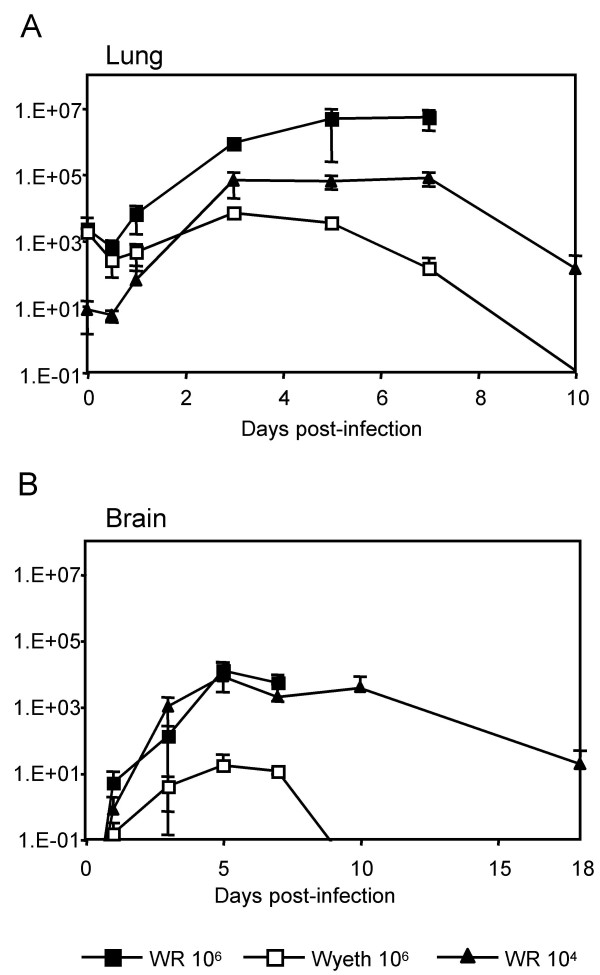
**Virus titers in the lungs (A) and the brains (B) of the infected mice**. Groups of three to five mice were infected with 10^6 ^(■) and 10^4 ^(▲) p.f.u. of WR and 10^6 ^p.f.u. (□) of Wyeth intranasally for each time point, and their lungs and brains were analyzed at various time points post-infection. Titers were calculated as p.f.u./mg tissue for the lung and the brain. The error bars indicate the standard deviations. The data of titers in lungs at 18 days post-infection was under detection level (not plotted in graph A).

In the lung (Fig. 2A), lethal infection with the WR strain (10^6 ^p.f.u.) resulted in a rapid increase of virus titer at 1 day post-infection and the virus titer continued to increase until mice died at 7–8 days post-infection. In non-lethal infection with the WR strain (10^4 ^p.f.u.), the virus titer in the lung increased at 1 day post-infection, reached maximum at 3 days post-infection, started to decline 7 days post-infection, and the virus was not detected at 18 days post-infection. In mice infected with Wyeth strain (10^6 ^p.f.u.), the virus titer in the lung also reached the maximum at 3 days post-infection, but the titer was much lower, and virus was cleared earlier.

The virus titers in the brain showed the same kinetics for the lethal infection with WR strain (10^6 ^p.f.u.) and the non-lethal infection with WR strain (10^4 ^p.f.u.) (Fig. 2B). It reached maximum at 5 days post-infection. In the non-lethal infection the virus titer was still high at 10 days post-infection, and the virus was not eliminated at 18 days post-infection, when mice were recovering their weight (Fig. 1A). Therefore, the virus titer in the brain is unlikely to be the sole determining factor of the lethality. In mice infected with the Wyeth strain (10^6 ^p.f.u.), virus titer in the brain also reached maximum at 5 days post-infection, but the titer was much lower, and the virus was cleared at 10 days post-infection (Fig. 2B).

### Virus replication in cultured mouse lung cells

**Figure 3:**
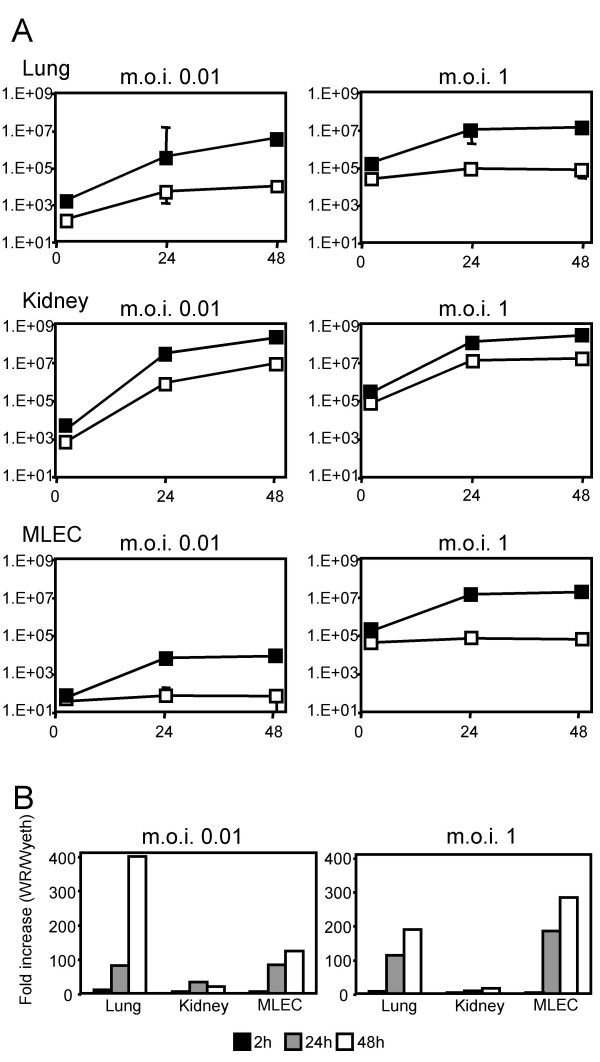
**Virus replications in primary mouse lung and kidney cells and MLEC after infection with WR and Wyeth strains at an m.o.i. of 0.01 and 1**. (A) Virus titers of WR (■) and Wyeth (□) strains in cells were determined at 2, 24 and 48 h post-infection. Titers are shown as p.f.u./well in log scale. The error bars indicate the standard deviations from triplicate samples. (B) Fold increase of WR titers compared to Wyeth titers at 2 (black bar), 24 (gray bar) and 48 (white bar) h post-infection.

In vivo data showed that the WR strain reached higher titers in the lung than the Wyeth strain (Fig. 2A), indicating that virus replication in lung cells were different between WR and Wyeth strains. To compare the replication of WR and Wyeth strains in lung cells, we examined virus replication in primary cultures of mouse lung cells. Primary kidney cells were also used for comparison. When these cells were infected at an m.o.i. (muliplicity of infection) of 0.01, both WR and Wyeth strains grew well (Fig. 3A). In lung cells, however, the WR strain grew about 400 times better than the Wyeth strain at 48 hours post-infection, whereas in kidney cells the difference was about 20 times (Fig. 3B). When mouse lung endothelial cells (MLECs) were infected, only the WR strain grew (Fig. 3A,B). In another set of experiments, to reduce the influence of cell-to-cell spread eliminate the influences of cell-to-cell spread, the cells were infected at an m.o.i. of 1, and similar results were obtained (Fig. 3A,B). These results show that the WR strain replicated more efficiently in lung cells and lung endothelial cells than the Wyeth strain.

**Figure 4:**
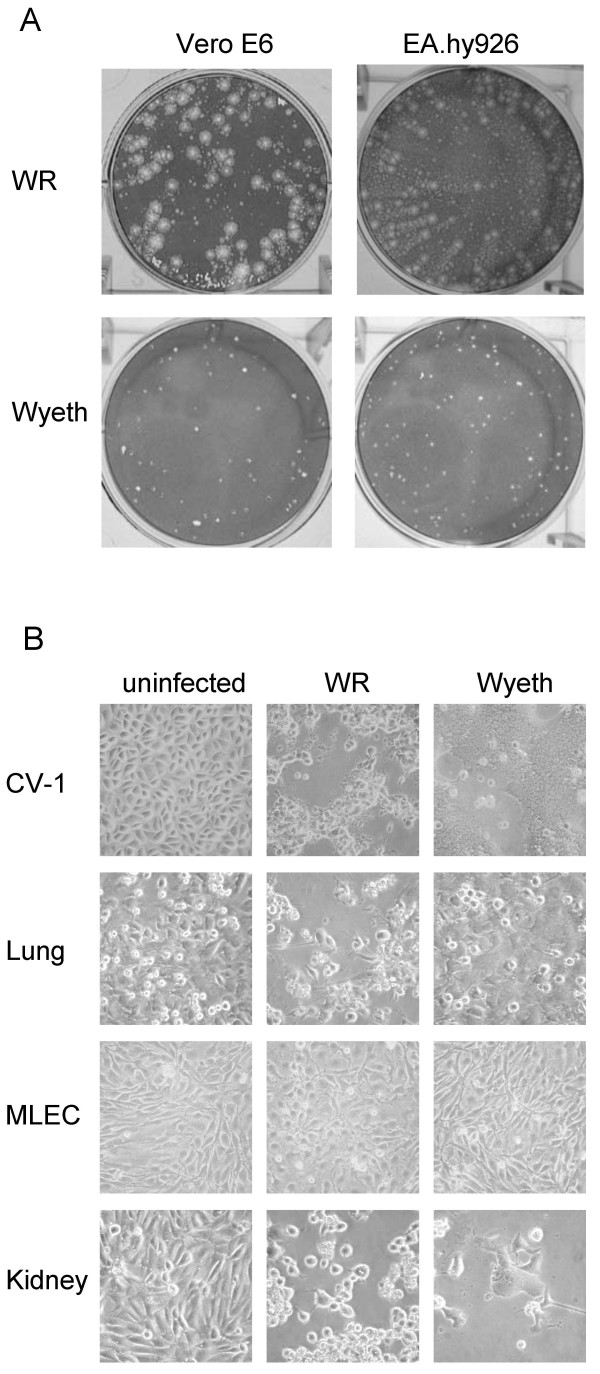
**Morphological changes of cell cultures infected with WR and Wyeth strains at 3 days post-infection**. (A) Plaque formation in Vero E6 and EA.hy926 cells. (B) Morphological changes of CV-1, primary mouse lung cells (Lung), MLEC and primary mouse kidney cells (Kidney) at 200 × magnification. Cells were infected with WR or Wyeth strains at m.o.i. 1 and observed after 2 days post-infection.

To examine cell-to-cell virus spread in more detail, we compared the plaque formation and the morphology of VV-infected cells. WR strain formed larger plaques and produced many comets on Vero E6 and EA.hy926 cell monolayers at 3 days post-infection (Fig. 4A). In contrast, the Wyeth strain formed smaller plaques and did not produce comets (Fig. 4A). The Wyeth strain induced syncytium formation in CV-1 cells, whereas the WR strain did not (Fig. 4B). These data suggest that cell-to-cell spread is also different between WR and Wyeth strains.

Wyeth strain did not alter the morphology of primary mouse lung cells, whereas the WR strain induced some cytopathic effect (CPE) (Fig. 4B). CPE was not apparent on MLECs infected by either strain (Fig. 4B). In mouse primary kidney cells, the WR strain induced typical CPE and the Wyeth strain formed syncytia similar to CV-1 cells (Fig. 4B). These observations suggest that there may be differences in the replication and dissemination in mouse lungs in vivo at an early phase of infection.

### Lymphocytes in the lung

Virus replication in the lung was suppressed at 5 days post-infection in mice inoculated with a non-lethal dose of WR and the Wyeth strains, while the virus titer in the lung continued to increase in mice infected with the lethal dose of WR until death of the mice (Fig. 2A). We analyzed immune responses at this early phase of the infection focusing on CD4^+ ^and CD8^+ ^T cells and NK cells.

**Figure 5:**
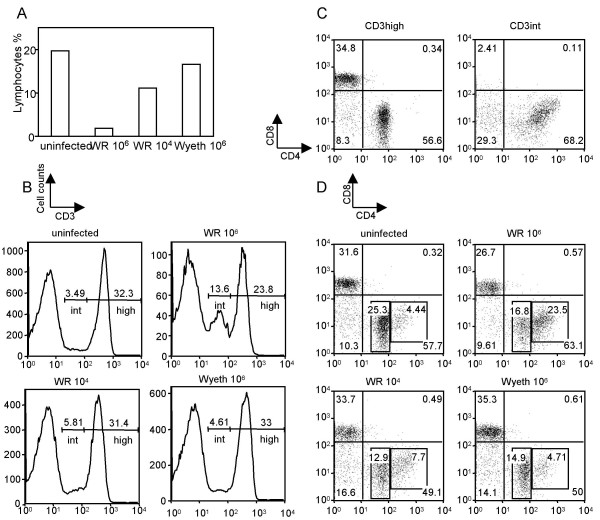
**Characterization of T lymphocytes in the lung at 5 days post-infection**. Mice were intranasally infected with 10^6 ^p.f.u. or 10^4 ^p.f.u. of WR or 10^6 ^p.f.u. of Wyeth strain. Cells were isolated from lungs and stained for T cell specific markers CD3, CD4 and CD8. (A) The percentage of lymphocytes in total lung cells isolated from uninfected control and infected mice. (B) Histograms of CD3 expression in lymphocytes from the lung. The percentages of CD3 intermediate (int) and CD3 high (high) subsets are shown in each histogram. (C) CD4 and CD8 expressions in CD3int and CD3high subsets. Data are representative of the lymphocyte subsets obtained from uninfected mice lungs. (D) CD4 and CD8 expressions in CD3 positive cells (CD3int and CD3high) from uninfected control and infected mice lungs. The percentages of CD4 high and CD4 intermediate are shown in each diagram.

At 5 days post-infection the proportion of lymphocytes in the total lung cells markedly decreased in mice given the lethal infectious dose of the WR strain compared to uninfected control mice (Fig. 5A). In contrast, non-lethal infection with WR and Wyeth strains only caused a mild reduction of lymphocytes (Fig. 5A). There were no differences in the percentages of the CD3^+ ^population (31–37%) of lymphocytes among the uninfected controls and lethally or non-lethally infected mice (Fig. 5B). We, however, noticed that there were two subsets of CD3-positive cells that expressed CD3 at a high level (CD3^high^) or intermediate level (CD3^int^) (Fig. 5B). Lethal-infection with the WR strain increased the proportion of CD3^int ^up to 13.6% compared to 3.49% of the uninfected control group (Fig. 5B). Non-lethal infection with WR and Wyeth did not induce a significant increase of this subset (Fig. 5B). CD4 expression in the CD3^int ^cells was higher than in the CD3^high ^cells (Fig. 5C). Therefore, the majority of the CD3^int ^cells were CD4^high ^CD8^- ^cells, whereas CD3^high ^cells consisted of CD4^int ^CD8^- ^and CD4^- ^CD8^+ ^cells (Fig. 5C). As the result, a proportion of CD3^int ^CD4^high ^CD8^- ^lymphocytes markedly increased in lethal-infection with the WR strain (23.5%) compared to uninfected controls (4.4%) at 5 days post-infection (Fig. 5D).

**Figure 6:**
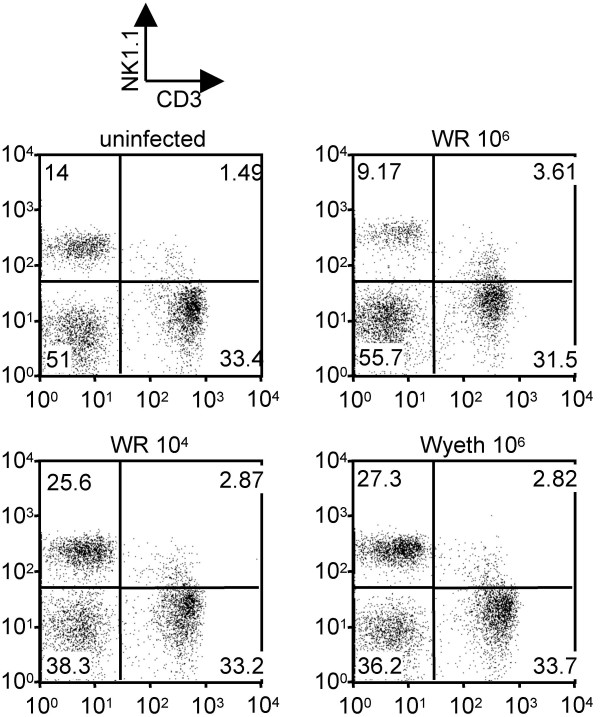
**Characterization of infiltrating NK cells in the lung at 5 days post-infection**. Mice were intranasally infected with 10^6 ^p.f.u. or 10^4 ^p.f.u. of WR or 10^6 ^p.f.u. of Wyeth. Cells isolated from lungs were stained with mAbs for NK1.1 and CD3.

The percentage of NK (NK^+^CD3^-^) lymphocytes in the lung declined (9.17%) in lethal infection at 5 days post-infection compared to the uninfected control (14%), whereas non-lethal infection increased the proportion of NK cells (25.6% and 27.3%) (Fig. 6).

These results indicate that the decrease of lymphocytes including T cells and NK cells and the alterations of the T cell phenotypes in the lung at the early phase of infection may be the reasons why virus replication was not suppressed in the lungs of mice with the lethal infection by the WR strain.

### Atrophy of thymus in mice inoculated with the WR strain

**Figure 7:**
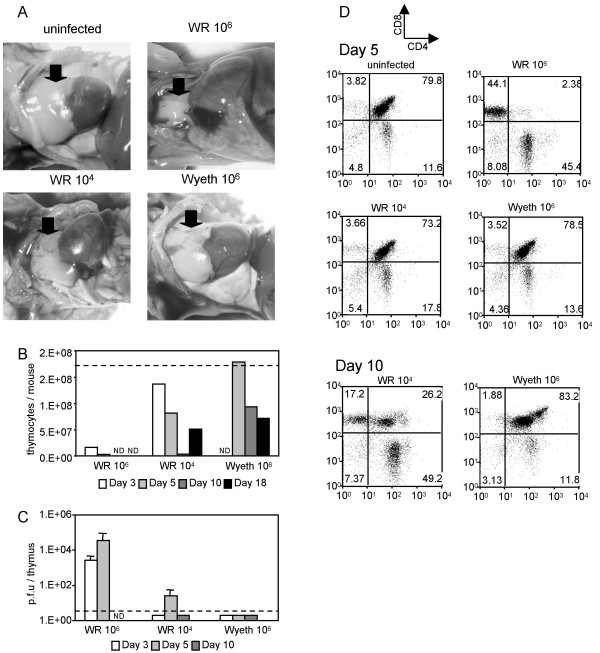
**Thymus atrophy in mice infected with WR and Wyeth strains**. (A) Pictures of the thymus in mice infected intranasally with 10^6 ^p.f.u. or 10^4 ^p.f.u. of WR or 10^6 ^p.f.u. of Wyeth at 5 days post-infection. Arrows indicate the thymi. (B) The numbers of thymocytes from mice infected with WR and Wyeth strains at 3 (white bar), 5 (light gray bar), 10 (dark gray bar) and 18 (black bar) days post-infection. Thymocyets were pooled from groups of three mice and counted. Data are shown as the average numbers of thymocytes per mouse. The dotted line is the average number of thymocytes from uninfected mice. ND; not determined. (C) Virus titers in thymus of mice infected with WR and Wyeth strains at 3 (white bar), 5 (light gray bar), and 10 (dark gray bar) days post-infection. Titers are shown as p.f.u. per organ in log scale. The error bars indicate the standard deviations in triplicate samples. The dotted line indicates the detection limit. ND; not determined. (D) CD4 and CD8 expressions of thymocyets from mice infected with WR and Wyeth strains at 5 (upper five panels) and 10 (lower two panels) days post-infection.

In addition to the decreased lymphocytes in the lung, intranasal inoculation with a lethal dose of WR strain induced severe thymus atrophy in C57BL/6J mice (Fig. 7A). The number of thymocytes decreased dramatically by 3 days post-infection compared to uninfected mice (Fig. 7B). This reduction was associated with a loss of CD4^+^CD8^+ ^DP cells (Fig. 7D). Non-lethal infection with WR reduced the size of the thymus to some extent (Fig. 7A). The number of thymocytes decreased to about a half of the thymocytes in the uninfected mouse at 5 days post-infection, reached a nadir at 10 days post-infection, and then started to recover (Fig. 7B). The proportion of CD4^+^CD8^+ ^DP cells was not different at 5 days post-infection (73.2%), but dropped to 26% at 10 days post-infection (Fig. 5D). The degree of the decrease of CD4^+^CD8^+ ^DP cells in the non-lethal infection was not as dramatic as in the lethal infection. Infection with the Wyeth strain did not change the size of the thymus (Fig. 7A) or the number of thymocytes by 5 days post-infection (Fig. 7B). The number of thymocytes started to decline after that (Fig. 7B), but the proportion of CD4^+^CD8^+ ^DP cells stayed unchanged (Fig. 7D).

Virus was recovered from the thymi of mice infected with the lethal dose of WR at 3 and 5 days post-infection (Fig. 7C). In contrast, the virus titers in the thymi in non-lethal infection were mostly below detection levels (Fig. 7C).

## Discussion

In this study, we analyzed virus replication and host immune responses in lethal and non-lethal VV infections in mice. We used two VV strains; the virulent WR strain and the less virulent Wyeth strain. Infection of C57 BL/6 mouse with a high dose (10^6 ^p.f.u.) of the WR strain was lethal, whereas a high dose (10^6 ^p.f.u.) of the Wyeth strain or a low dose (10^4 ^p.f.u.) of the WR strain were not lethal. Replication of virus in the lung, but not in the brain, correlated with lethality. The WR strain is known to cause encephalitis after intracranial inoculation [22,25]. We, however, did not observe neurological symptoms in any of the infected mice, and think it unlikely that encephalitis caused death of the mice infected with a high dose of the WR strain by the respiratory route.

The lethal infection with WR strain was associated with a decrease of the number of T cells and NK cells in the lung and with an altered phenotype of T cells compared to uninfected controls and non-lethal infections. We used isoflurane to anesthetize mice before intranasal inoculation for consistent administration of the inoculum into the airway. Isoflurane is known to inhibit interferon stimulation of NK cells in mice [26,27]. It was also reported that mean white blood cell counts in circulation decreased in mice after exposure to isoflurane anesthesia [28]. In our experiments uninfected control mice were also anesthetized with isoflurane before inoculation of CV-1 cell lysate. Therefore, we interpret the observed decrease of the number of T cells and NK cells in the lung of lethally infected mice (when compared to uninfected controls and non-lethal infections) is due to viral infection, although synergistic effect of viral infection and isoflurane cannot be ruled out.

The lethal infection with WR strain also induced severe thymus atrophy resulting in the reduction of CD4^+^CD8^+ ^DP T cells in the thymus. These data suggest that lethal infection with the WR strain by the respiratory route induces immune suppression, resulting in uncontrolled virus replication in the lung. Innate immune responses are likely to be very important to limit virus replication in the lung in the early phase of the infection (1–3 days post-infection) for subsequent recovery from VV infection. In the absence of effective early immune responses, the mice infected with a high dose of WR do not develop effective adaptive immune responses and the high viral burden suppresses the T cell responses in the lungs and causes severe thymic atrophy.

Intranasal infection with VV resulted in peak virus titers in the lung at 3–7 days post-infection (Fig. 2). This replication pattern is similar to infection with paramyxoviruses such as respiratory syncytial virus (RSV) and pneumonia virus of mice (PVM), in which virus titers in the lung peak at 4–5 days post-infection [29-31]. In contrast, other respiratory viruses, such as influenza and SARS viruses, rapidly replicate in the lung after intranasal infection and the virus titers reached a peak at 1–2 days post-infection [32-34]. Although RSV has direct cytopathic effects on the respiratory epithelium, host immune responses are more important factors in disease pathogenesis [35]. In lethal intranasal infection with VV, in contrast, there is a marked decrease in lymphocytes in the lung. T cells are key effectors of virus clearance in mice infected with various viruses via respiratory route [36]. Immune suppression of T cells is considered to be an immune evasion strategy, observed in several respiratory virus infections. For example, PVM and RSV suppressed T cell effecter functions in the lung [37,38]. Adenovirus infection induced T cells with decreased proliferative ability in the lung and local immunoincompetence by altering DC-T-cell interaction [39]. Infection with highly virulent influenza A strains down-regulated CD8^+ ^T cell responses [33]. Since VV has many immunomodulatory proteins [40-42], it is not surprising that high levels of WR VV induced immune suppression in the infected lungs.

We observed that intranasal VV infection induced severe thymus atrophy (Fig. 7). Severe atrophy of the thymus is seen in several infections [43] including acute viral infections, such as rabies [44,45], measles [46,47], mouse hepatitis [48], and Ebola viruses [49]. Although inoculation of ectromelia virus into the food-pad of BALB/c mouse is known to cause the necrosis of thymus as well as other lymphatic tissues [50], there has been no description of thymus atrophy in mice induced by respiratory infection with VV. It is not known whether RSV, PVM, adenovirus and influenza A virus cause thymus atrophy when immune suppression of T cells is observed in the lung. The atrophy of the thymus was mainly due to a reduction of immature CD4^+^CD8^+ ^DP. One major consideration regarding thymus atrophy is a rise of glucocorticoid hormone level in the blood, which is induced due to the stress responses to severe infections [43,44,51,52]. CD4^+^CD8^+ ^DP thymocytes are particularly sensitive to glucocorticoids. In addition, some cytokines, such as tumor necrosis factor-α, may also contribute to the thymus atrophy in some infections [48,53]. It also has been suggested that infection of the cells in thymus is involved in reducing the number of thymocytes [43,46-48,54,55]. In measles and mouse hepatitis virus infections apoptotic depletion of thymocytes is mediated by the infection of thymic epithelial cells [46-48]. In Trypanosoma cruzi infection parasite-derived factors are involved in apoptosis of thymocytes [43,54,55]. VV has been found to synthesize steroid hormones with a 3β-hydroxysteroid dehydrogenase encoded by gene A44L [56]. We did not perform experiments dissecting the mechanisms of the thymus atrophy induced by intranasal VV infection in the lethal infection. However, virus was recovered from the thymus when the thymocyte number was markedly decreasing at days 3 and 5 post-infection in the lethal infection (Fig. 7B and 7C), suggesting that the steroid hormone synthesized by both virus and host may contribute to thymus atrophy as well as the direct effect of virus infection against thymic cells. The VV A44L gene is well conserved among VV strains. In contrast, virus was under the detection limit in the thymi of mice infected with a non-lethal dose of WR when the thymocyte number was at nadir at day 10 post-infection (Fig. 7B and 7C). Further investigation is required to uncover the mechanisms of thymus atrophy induced by respiratory infection with VV. At this point it appears that acute thymus atrophy early in infection may be a component of the lethal outcome.

WR and Wyeth strains differed in the ability to grow in primary cultures of lung and lung endothelial cells, which reflected the in vivo growth of these viruses in the lung. The size of the virus plaques and the ability to form comets were also different between these two strains (Fig. 4A). In addition, the Wyeth strain induced syncytium formation in infected cells, but the WR strain did not (Fig. 4B). Usually cell-cell fusion is not apparent during VV infection in cultured cells, but it appears spontaneously with certain mutants or can be triggered by briefly lowering the pH [57]. These differences suggest different host-virus interactions in infected lung cells, and in the case of WR infection, a high dose of virus resulted in uncontrolled replication of the virus in the lung. It has been reported that the hemagglutin and serine protease inhibitor 3 are fusion inhibitor proteins and mutants containing a disruption of these proteins form syncytia at neutral pH [58-62]. We did not analyze nucleotide differences between WR and Wyeth strains, since the Wyeth strain is not cloned and is genetically heterogeneous, which recent sequencing data of Dryvax (derived from the same New York City Board of Health strain) confirmed (GenBank accession numbers DQ377945, AY313847, AY313848).

## Conclusion

This study suggests that the lethality induced by intranasal infection with a high dose of the WR strain of VV is caused by increased replication of the virus in lung cells and lymphocyte depletion in the lungs and the thymus during the early phase of the infection, resulting in uncontrolled virus replication in the lung. Murine models of respiratory infection with several viruses have been provided useful information about acute and chronic virus respiratory infection, humoral and cellular immune responses, CD4 and CD8 T cell biology, and innate immune responses. We think that VV respiratory infection of the mouse is a useful model not only to understand the pathogeneses of poxviruses but also other respiratory viruses.

## Methods

### Viruses and cells

Vaccinia virus WR strain was kindly provided by Girish J. Kotwal, Division of Medical Virology, University of Cape Town, Cape Town, South Africa, and William L. Marshall of Department of Medicine, University of Massachusetts Medical School, and the Wyeth strain was kindly provided by Margo A. Brinton of the Department of Biology, Georgia State University, Atlanta, GA, through Ching-Juh Lai of Laboratory of Infectious Diseases, National Institute of Allergy and Infectious Diseases, National Institutes of Health. Viruses were grown in CV-1 cells (ATCC CCL-70) and virus stocks were prepared from cell lysates. CV-1 cells were maintained in Minimum Essential medium (MEM) (Invitrogen, Carlsbad, CA) containing 10% fetal bovine serum (FBS). A human endothelial cell line, EA.hy926, was kindly provided by Cora-Jean S. Edgell, University of North Carolina. EA.hy926 cells and Vero E6 cells (ATCC CRL-1586) were maintained in Dulbecco's modified Eagle's medium (DMEM) (Invitrogen) containing 10% FBS. Virus titers were determined by plaque forming assay using CV-1 cells following the standard procedure [63].

### Isolation of mouse primary lung and kidney cells

Primary mouse lung and kidney cells were isolated from female C57BL/6J mice. Lungs and kidneys were removed and diced with scissors. Then, each organ was digested with 1 mg/ml collagenase A (Roche Diagnostics, Indianapolis, IN) for 45 min, filtered with a 70 μm cell strainer (BD Biosciences, Bedford, MA), and the separated cells were grown in DMEM supplemented with 10% FBS.

MLECs were isolated and grown as previously reported [64,65]. Briefly, mouse lung cells were incubated with Dynabeads^® ^M-450 Sheep anti-Rat IgG (Dynal Biotech ASA, Oslo, Norway) coated with MEC13.3 (anti-mouse CD31 rat IgG)(BD PharMingen, San Diego, CA). CD31-expressing cells were separated by Dynal MPC-L Magnetic Particle Concentrator (Dynal Biotech ASA, Oslo, Norway) and grown in a gelatin-coated tissue culture flask. One week later, adherent cells were trypsinyzed, and CD102-expressing cells were isolated by 3C4 (anti-mouse CD102 rat IgG)(BD PharMingen)-coated beads and grown in DMEM with 20% FBS supplemented with 100 μg/ml heparin sodium salt from porcine intestinal mucosa (Sigma-Aldrich, St. Louis, MO) and 100 μg/ml endothelial cell growth supplement (BD Biosciences). Cells were confirmed to express the endothelial specific markers, PECAM (CD31), ICAM-2 (CD102) and VE-cadherin (CD144) by flow cytometric analysis.

### Virus inoculation

4–6 week-old female C57BL/6J mice were purchased from The Jackson Laboratory. Mice were anesthetized with isoflurane and then intranasaly inoculated with various doses (10^3 ^to 10^6 ^p.f.u.) of the WR or Wyeth strains, or uninfected CV-1 cell lysate (for control mice) in a total volume of 50 μl. Mice were observed for disease symptoms and weighed daily. All mice were maintained in the Animal Facility at the University of Massachusetts Medical School, which is regulated by AWA-1995, PHS-1986, MA140-1985 and follows the AAALAC-1965 guidelines.

### Virus titration in organs

Lungs, brains and thymi were taken from mice inoculated with VV at different days post-infection and kept frozen at -80°C until use. Each organ was ground on a 40 μm cell strainer (BD Biosciences) in ten volumes of phosphate-buffered saline (PBS) with 10% FBS and sonicated for 30 seconds six times. Virus titers were determined by plaque forming assays and titers were calculated as p.f.u./mg tissue for the lung and the brain, and p.f.u./total tissue for the thymus.

### Virus growth curve in cell culture

CV-1, Vero E6, primary mouse lung and kidney cells, and MLECs were grown in 6-well plates until cells became confluent. The monolayers were inoculated with VV for 2 hours at indicated multiplicity of infection (m.o.i.). Cells were harvested at different time points after infection and kept at -80°C until use. Cells were resuspended in 100 μl of PBS with 10% FBS and sonicated. Virus titers were determined by plaque forming assay using CV-1 cells and titers were calculated as p.f.u./well.

### Recovery of leukocytes from lungs, thymi and spleens

Lung leukocytes were isolated from VV-infected mice and uninfected control mice at indicated time points. Lungs were inflated with RPMI medium (Invitrogen) through the trachea and washed with PBS. A pool of lungs from four mice was minced and digested with collagenase A (Roche Diagnostics), and then lung cells were strained with a 70 μm cell strainer (BD Biosciences). Live cells were separated from dead cells or debris of lung tissue by centrifugation with Ficoll-Paque (GE Healthcare Bio-sciences AB, Uppsala, Sweden). Thymocytes were also recovered from VV-infected mice and uninfected control mice at indicated time points. A pool of thymocytes or splenocytes from four mice was lysed with RBC lysis buffer (Sigma-Aldrich), and resuspended in RPMI medium.

### Flow cytometric analysis of cell-surface antigens

Lung leukocytes and thymocytes were washed and were blocked with Purified Rat Anti-Mouse CD16/CD32 (FCγIII/II Receptor) Monoclonal Antibody (Mouse BD Fc Block)(BD PharMingen) in FACS buffer (PBS containing 0.1% BSA and 0.1% sodium azide). Cells were stained with a mixture of different fluorescent-labeled antibodies directed at surface phenotypic markers, CD45, CD3, CD4, CD8a, NK1.1 and CD25 (eBioscience, San Diego, CA), and then fixed with 2% paraformaldehyde. The stained cells were analyzed by FACSAria (BD Biosciences). Leukocytes were recognized by characteristic size (forward scatter, FSC), granularity (side scatter, SSC) and CD45 expression.

## Competing interests

The author(s) declare that they have no competing interests.

## Authors' contributions

DH, FAE and MT conceived of the study. DH designed and performed the experiments. DH and MT analyzed the results. DH, FAE and MT discussed the results and prepared the manuscript. All authors read and approved the final manuscript.
